# One-year morbidity and mortality in patients treated with standard-dose and low-dose apixaban after acute large vessel occlusion stroke

**DOI:** 10.1007/s11239-024-02954-7

**Published:** 2024-03-31

**Authors:** Yasutaka Murakami, Kenichi Todo, Kazutaka Uchida, Hiroshi Yamagami, Nobuyuki Sakai, Yasufumi Gon, Shuhei Okazaki, Tsutomu Sasaki, Shinichi Yoshimura, Takeshi Morimoto, Hideki Mochizuki

**Affiliations:** 1 Department of Neurology, Osaka University Graduate School of Medicine, 2-2 Yamadaoka, Suita, Osaka 565-0871 Japan; 2 Department of Neurosurgery, Hyogo Medical University, Nishinomiya, Hyogo Japan; 3 Department of Stroke Neurology, National Hospital Organization Osaka National Hospital, Osaka, Japan; 4 Department of Neurosurgery, Kobe City Medical Center General Hospital, Nishinomiya, Hyogo Japan; 5 Department of Clinical Epidemiology, Hyogo Medical University, Nishinomiya, Hyogo Japan

**Keywords:** Large vessel occlusion, Atrial fibrillation, Apixaban, Dose-reduction criteria

## Abstract

Although low-dose direct oral anticoagulants (DOACs) are recommended for patients at high risk of bleeding complications, it remains unclear whether the dose reduction in real-world setting is also appropriate in patients after large-vessel occlusion (LVO) stroke. This study hypothesized that patients with atrial fibrillation (AF) and LVO receiving low-dose DOACs have an increased risk of ischemic and hemorrhagic events. The study aimed to assess 1 year morbidity and mortality in patients treated with standard-dose and low-dose apixaban after LVO stroke. A post hoc analysis was performed using the acute LVO registry data, which enrolled patients with AF and LVO who received apixaban within 14 days of stroke onset. The incidences of ischemic events (ischemic stroke, acute coronary syndrome, acute myocardial infarction, and systemic embolism), major bleeding events, and death from any cause were compared between patients receiving standard- and low-dose apixaban. Of 643 patients diagnosed with LVO, 307 (47.7%) received low-dose apixaban. After adjustment for clinically relevant variables, no significant differences were observed in the incidence of ischemic events (adjusted hazard ratio [aHR]: 2.12, 95% confidence interval [CI] 0.75–6.02), major bleeding events (aHR: 1.17, 95% CI 0.50–2.73), and death from any cause (aHR: 1.95, 95% CI 0.78–4.89) between patients receiving standard- and low-dose apixaban. No significant differences were observed in the incidence of ischemic events, major bleeding events, or death from any cause between patients with AF and LVO receiving standard- and low-dose apixaban.

**Supplementary Information:**

The online version contains supplementary material available at 10.1007/s11239-024-02954-7.

## Highlights

- It is unclear whether the dose reduction in real-world setting is appropriate in patients after large-vessel occlusion (LVO) stroke.
- This study showed that the risk of ischemic/bleeding events or mortality is not significantly increased in patients receiving low-dose apixaban after LVO stroke compared with those receiving standard-dose apixaban.
- It is probable that the background factors, rather than the apixaban dose, were probably responsible for the differences in mortality outcomes in patients with atrial fibrillation and LVO.
- Studies targeting other DOACs should be considered because each DOAC has different criteria for dose adjustment.

## Introduction

Apixaban, a direct Factor Xa inhibitor, was reported to be effective and safe for preventing stroke in patients with atrial fibrillation (AF) compared with warfarin in a large randomized clinical trial (ARISTOTLE) [1]. The standard dose of apixaban is 5 mg twice daily. However, it is recommended that the dose be reduced to 2.5 mg twice daily in patients at high risk of bleeding complications who meet at least two of the following three criteria: (i) age ≥ 80 years, (ii) weight ≤ 60 kg, and (iii) serum creatinine ≥ 1.5 mg/dL. In the ARISTOTLE study, only 4.7% of patients received low-dose apixaban [1]; while 30–63% of patients received low-dose apixaban in recent real-world observational studies [2–8]. However, several studies have reported a higher incidence of cardiovascular events and mortality in patients receiving low-dose apixaban than in those receiving standard-dose apixaban although these results were attributed to background factors such as age [2–5].

On the other hand, a previous study showed that patients with AF and a large ischemic stroke had an increased risk of recurrent stroke and major bleeding compared with those with a small ischemic stroke [9]. One possible reason is that the current dose adjustment for direct oral anticoagulants (DOACs) in real-world clinical setting may be inappropriate for patients at high risk of recurrent stroke such as patients with large ischemic stroke or large-vessel occlusion (LVO). Therefore, this study hypothesized that patients receiving low-dose DOACs would have an increased risk of ischemic and hemorrhagic events in patients after LVO stroke, even after adjusting for background factors. This study aimed to assess the 1 year morbidity and mortality by comparing the incidence of ischemic events (ischemic stroke, acute coronary syndrome, acute myocardial infarction, and systemic embolism), major hemorrhagic events, and death from any cause of patients receiving standard- and low-dose apixaban in a large Japanese registry of patients with acute cardioembolic stroke and LVO.

## Methods

### Participants and data collection

This study was a post hoc analysis of the Apixaban on clinical outcome of the patients with large vessel occlusion or stenosis (ALVO) study. The inclusion criteria of ALVO study were patients aged at least 20 years, with acute ischemic stroke with LVO or intra-/extra-cranial artery stenosis and AF, and received apixaban within 14 days after the onset [10]. The definition of AF in ALVO trial did not include patients with mechanical valves or moderate-to-severe mitral stenosis, who were ineligible for the use of DOACs. In principle, apixaban was administered according to medical package insert in Japan, and low-dose apixaban was used for patients with at least two of the following three criteria: (i) age ≥ 80 years, (ii) weight ≤ 60 kg, and (iii) serum creatinine ≥ 1.5 mg/dL; however, the actual dosage was selected by the treating physicians. The exclusion criteria were patients who are considered ineligible for the study by the investigator, pregnant or potentially pregnant, with a history of hypersensitivity to apixaban, with liver disease with coagulopathy and clinically significant bleeding risk, with renal insufficiency (creatinine clearance < 15 mL/min) and with pathological bleeding including intracranial bleeding of any type [10]. Clinical information was collected by reviewing medical records. Follow-up information was collected at 30, 90, and 365 days of stroke onset, and any additional information was obtained by contacting the patients, relatives, and referring physicians.

This study excluded patients with stenosis without LVO. LVO defined as (i) M1—3 segment middle cerebral artery occlusion, (ii) A1—2 segment anterior cerebral artery occlusion, (iii) P1—2 segment posterior cerebral artery occlusion, (iv) internal carotid artery occlusion, (v) basilar artery occlusion, or (vi) vertebral artery occlusion were included. The following baseline data were used in the analyses: age, sex, underlying disease (hypertension, diabetes mellitus, chronic heart failure, a history of ischemic stroke, cerebral hemorrhage, subarachnoid hemorrhage, and coronary artery disease), post-stroke CHD_2_DS_2_-VASc score [11], pre-stroke modified Rankin Scale (mRS) score [12], antithrombotic medication at stroke onset, National Institute of Health Stroke Scale (NIHSS) score [13], serum creatinine, blood glucose and HbA1c on admission, occlusion site, use of intravenous recombinant tissue plasminogen activator (IV rt-PA; 0.6 mg/kg), endovascular therapy (EVT) using any device approved in Japan, and modified thrombolysis in cerebral infarction (mTICI) score for patients with EVT [14]. The body weight data were not collected in the ALVO study, which primarily planned to confirm the safety of early administration of apixaban. The presence of intracranial hemorrhage prior to apixaban administration was also evaluated using the Heidelberg Bleeding Classification [15].

### Study end points

Ischemic events, major bleeding events, and death from any cause were assessed within 365 days of apixaban administration. The ischemic events included ischemic stroke, acute coronary syndrome, acute myocardial infarction, and systemic embolism. Major bleeding events were defined as any bleeding event according to the International Society on Thrombosis and Haemostasis major bleeding [16].

### Statistical analysis

Patient characteristics and outcomes were compared between those who received standard-dose apixaban (5 mg twice daily) and those who received low-dose apixaban (2.5 mg twice daily). Values are presented as mean ± standard deviation or median with interquartile range (IQR) for continuous variables and as numbers and percentages for categorical variables. Continuous variables were compared using Student’s *t*-test or Wilcoxon rank-sum test based on the distributions. Fisher’s exact or chi-squared tests were used for categorical variables when appropriate. The cumulative incidences of ischemic events, major bleeding events, and death from any cause were analyzed. The starting point of the follow-up study was the time of apixaban administration. Patients were censored at the time of ischemic events, major bleeding events, or death within 365 days of index stroke onset.

Univariate Cox regression models were developed to analyze the associations between baseline variables and outcomes. Multivariate Cox regression models were developed to assess the independent association between the apixaban dose and outcome, adjusting for the following clinically relevant variables: age, sex, hypertension, diabetes mellitus, chronic heart failure, history of ischemic stroke, prior antiplatelet therapy, serum creatinine level, IV rt-PA, and EVT. To assess the heterogeneity of the association between apixaban dose and outcome by baseline characteristics, subgroup analyses based on sex, age (≥ 80 or < 80), with or without IV rt-PA, and with or without EVT were performed. The interactions were tested using a multiplicative interaction term (each outcome × variable) included in the models.

Sensitivity analyses were performed to validate the results. Multivariate stepwise Cox regression models were developed to reduce the number of confounders; these models included parameters associated with each outcome in the univariate analysis (*p* < 0.05). All statistical analyses were performed using EZR version 1.55 (Saitama Medical Centre, Jichi Medical University, Saitama, Japan) [17], a graphical user interface for R (version 4.1.3; R Foundation for Statistical Computing, Vienna, Austria). All reported p*-*values were two-tailed, and statistical significance was set at p < 0.05.

## Results

### Patient characteristics

**Fig. 1:**
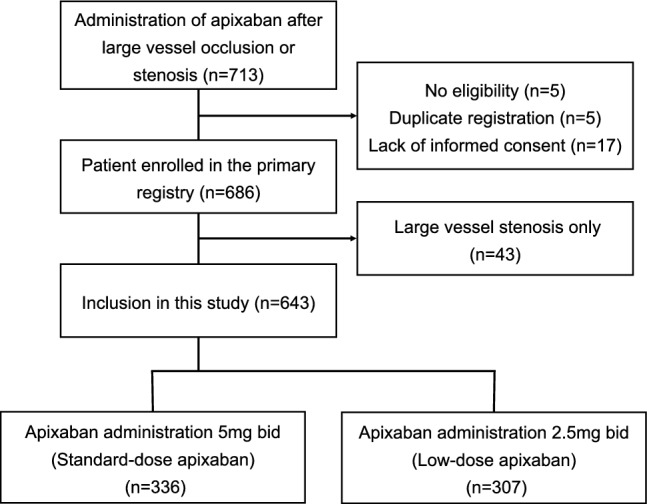
Study flow chart of the 713 registry participants, 27 were excluded from the primary registry. Patients without LVO were also excluded (n = 43), and 643 patients were finally enrolled in the study

Of the 713 registry participants, 27 were excluded from the primary multicenter study. Patients without LVO were excluded (n = 43), and 643 patients were enrolled in the present study (Fig. 1). Standard- and low-dose apixaban were prescribed to 336 and 307 patients, respectively. The median follow-up period was 364 (IQR: 347–365) days for patients on standard-dose apixaban and 358 (IQR: 170–365) days for patients on low-dose apixaban (*p* < 0.001). The baseline characteristics are shown in Table 1. Compared to patients on standard-dose apixaban, those on low-dose apixaban were significantly older (71.5 ± 8.5 vs. 83.6 ± 6.7 years, *p* < 0.001), more likely to be female (31.0% vs. 66.4%, *p* < 0.001), and had a higher prevalence of previous ischemic stroke (13.4% vs. 20.2%, *p* = 0.026) and chronic heart failure (3.6% vs. 10.2%, *p* = 0.001). Consistent with the above findings, the low-dose apixaban group had higher post-stroke CHA_2_DS_2_-VASc scores than the standard-dose group (median [IQR]: 4 [3–5] versus 5 [4–6], *p* < 0.001).

**Table 1:** Patient characteristics

Characteristics	Standard dose (n = 336)	Low dose (n = 307)	*p* value
Age (years), mean (SD)	71.7 (8.5)	83.6 (6.7)	< 0.001
Female, n (%)	104 (31.0)	204 (66.4)	< 0.001
History of ischemic stroke, n (%)	45 (13.4)	62 (20.2)	0.026
History of cerebral hemorrhage, n (%)	3 (0.9)	6 (2.0)	0.32
History of subarachnoid hemorrhage, n (%)	3 (0.9)	1 (0.3)	0.63
History of coronary artery disease, n (%)	12 (3.6)	7 (2.3)	0.36
Hypertension, n (%)	76 (22.9)	74 (24.4)	0.71
Diabetes mellitus, n (%)	20 (6.0)	17 (5.6)	0.87
Chronic heart failure, n (%)	12 (3.6)	31 (10.2)	0.001
CHA_2_DS_2_-VASc, median (IQR)	4 (3–5)	5 (4–6)	< 0.001
mRS before onset 0–1, n (%)	306 (91.1)	209 (68.1)	< 0.001
Prior antiplatelet, n (%)	53 (15.8)	71 (23.1)	0.021
Prior anticoagulant, n (%)	75 (22.3)	77 (25.1)	0.46
NIHSS score, median (IQR)	14 (7–18)	14 (8–21)	0.058
Anterior circulation stroke, n (%)	235 (69.9)	229 (74.6)	0.22
Laboratory data			
Blood glucose, mg/dL, mean (SD)	130.1 (45.4)	129.6 (34.4)	0.28
Creatinine, mg/dL, mean (SD)	0.85 (0.26)	0.85 (0.35)	0.08
HbA1c (NGSP), %, mean (SD)	6.1 (0.7)	6.0 (0.7)	0.99
Initial treatment			
IV rt-PA, n (%)	158 (47.0)	106 (34.5)	0.001
EVT, n (%)	202 (60.1)	157 (51.1)	0.026
TICI2b/3 (only EVT), n (%)	193 (95.5)	136 (87.2)	< 0.001
IV rt-PA and/or EVT, n (%)	246 (73.2)	183 (59.6)	< 0.001
Cerebral hemorrhage before apixaban initiation, n (%)	62 (18.4)	48 (15.6)	0.35
Days from onset to apixaban initiation, mean (SD)	3.5 (3.0)	4.0 (3.4)	0.062

*ACA* anterior cerebral artery, *BA* basilar artery, *EVT* endovascular therapy *NIHSS* national institute of health stroke scale, *ICA* internal carotid artery, *IQR* interquartile range, *IV rt-PA* intravenous recombinant tissue plasminogen activator, *mRS* modified rankin scale; *M1* the horizontal segment of the middle cerebral artery, *M2* the insular segment of the middle cerebral artery, *PCA* posterior cerebral artery, *SD* standard deviation, *VA* vertebral artery

Intravenous thrombolysis and EVT were performed more frequently in the standard-dose apixaban group than in the low-dose apixaban group (IV rt-PA, 47.0% vs. 34.5%, *p* = 0.001; EVT, 60.1% vs. 51.1%, *p* = 0.026). Effective endovascular revascularization, defined as mTICI 2b or 3, was observed more frequently in the standard-dose group than in the low-dose group (95.5% vs. 87.2%, *p* < 0.001). The median duration from onset to apixaban administration was 3.5 ± 3.0 days in the standard-dose group compared to 4.0 ± 3.4 days in the low-dose group (*p* = 0.062).

### Outcomes

**Fig. 2:**
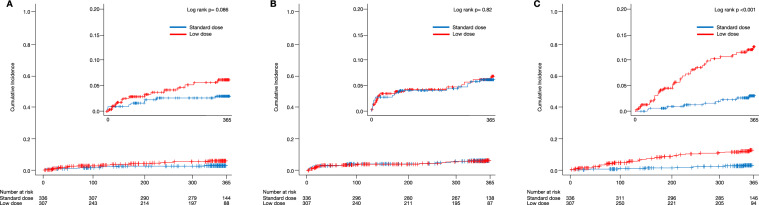
Kaplan–Meier curves for events in patients on standard- and low-dose apixaban. Image showing the cumulative incidence of the ischemic events (**A**), major bleeding events (**B**), and death from any cause (**C**). The inset of each panel shows the same data with the Y-axis enlarged

The cumulative incidence of ischemic events tended to be higher in patients receiving low-dose apixaban (3.0%/year vs. 6.7%/year, log-rank *p* = 0.086, Fig. 2a, Table 2). The crude hazard ratio (HR) for ischemic events in patients on low-dose apixaban was 2.06 (95% confidence interval [CI] 0.90–4.72). The adjusted HR for ischemic events in patients on low-dose apixaban was 2.12 (95% CI 0.85–5.25) after adjustment for clinically relevant variables (Table 2). In the subgroup analyses, low-dose apixaban was associated with an increased risk of ischemic stroke in patients with EVT; however, no interactions were observed in any subgroup analysis (Supplemental Table 1).

**Table 2:** Outcomes in patients administered standard-dose apixaban compared with those administered low-dose apixaban

	Standard dose	Low dose	Crude HR (95% CI)	*p* value	Adjusted HR (95% CI)	*p* value
	n (%/year)	n (%/year)				
Ischemic events	9 (3.0)	15 (6.7)	2.06 (0.90–4.72)	0.086	2.12 (0.75–6.02)	0.16
Major bleeding events	19 (6.6)	17 (7.6)	1.08 (0.56–2.07)	0.82	1.17 (0.50–2.73)	0.72
Death from any cause	9 (3.0)	32 (13.8)	4.55 (2.17–9.53)	< 0.001	1.95 (0.78–4.89)	0.15

The adjusted variables were age, sex, hypertension, diabetes mellitus, chronic heart failure, history of ischemic stroke, prior antiplatelet therapy, serum creatinine level, IV rt-PA, and EVT

The ischemic events included ischemic stroke, acute coronary syndrome, acute myocardial infarction, and systemic embolism. Major bleeding events included any bleeding event, according to the International Society on Thrombosis and Haemostasis major bleeding

*CI* confidence interval, *EVT* endovascular therapy, *HR* hazard ratio, *IV rt-PA* intravenous recombinant tissue plasminogen activator

Patients on standard-dose apixaban had a similar rate of major bleeding events as patients on low-dose apixaban (6.6%/year vs. 7.6%/year, log-rank *p* = 0.82, Fig. 2b, Table 2). Compared with the standard-dose apixaban group, the crude HR for major bleeding events in the low-dose apixaban group was 1.08 (95% CI 0.56–2.07). Additionally, the adjusted HR for major bleeding events in the low-dose apixaban group was 1.17 (95% CI: 0.50–2.73) after adjustment for clinically relevant variables (Table 2). No associations were observed between apixaban dose and major bleeding events in any subgroup analysis (Supplemental Table 2).

The cumulative incidence of death from any cause was significantly higher in the low-dose apixaban group than in the standard-dose group (3.0%/year vs. 13.8%/year, log-rank *p* < 0.001, Fig. 2c, Table 2). The crude HR for death from any cause in the low-dose apixaban group was 4.55 (95% CI: 2.17–9.53). However, after adjustment for clinically relevant variables, low-dose apixaban was no longer associated with death from any cause compared with standard-dose apixaban (adjusted HR: 1.95, 95% CI: 0.78–4.89; Table 2). In addition, subgroup analyses showed that low-dose apixaban was not associated with death from any cause when stratified according to age (80 years). (Supplemental Table 3).

In the sensitivity analyses, the adjusted HR for ischemic events in patients on low-dose apixaban was 1.95 (95% CI: 0.85–4.46, *p* = 0.12; Supplemental Table 4). For the bleeding events, the HR for patients on low-dose apixaban was 0.97 (95% CI 0.50–1.88, *p* = 0.92; Supplemental Table 5). For death from any cause, the HR in patients on low-dose apixaban was 2.00 (95% CI 0.81–4.93, *p* = 0.13; Supplemental Table 6). These results were consistent with those of the main analyses.

## Discussion

This study demonstrated that the incidence of ischemic events tended to be higher, the incidence of hemorrhagic events did not differ, and mortality from any cause was higher in patients with acute stroke treated with low-dose apixaban than in those treated with standard-dose apixaban. However, this difference disappeared when confounding factors were corrected. These results do not indicate an increased risk of ischemic/bleeding events or mortality in patients receiving low-dose apixaban after LVO stroke.

Several studies have compared the morbidity of standard-dose versus low-dose apixaban in patients with AF [2–5]. These studies concluded that ischemic and bleeding events were comparable or more likely to occur in patients receiving low-dose apixaban than in those receiving standard-dose apixaban. However, these results are considered to reflect the comorbidities of the patients, as low-dose apixaban was not associated with ischemic and bleeding events when adjusted for age and other factors. Similar results were observed in specific patient groups at a higher risk of ischemic and bleeding events. For example, in older persons or patients with a history of ischemic stroke, who are prone to developing ischemic and hemorrhagic events, no significant differences in ischemic and bleeding events were observed between patients taking standard- and low-dose apixaban [5, 18]. LVO leads to large ischemic lesions, and patients with large ischemic lesions are at a higher risk of recurrent stroke and major bleeding [9]. The present study showed that recurrent stroke and major bleeding were not significantly different between standard and low apixaban doses in patients with acute LVO.

Previous studies have reported that patients administered low-dose apixaban were associated with increased mortality compared to patients administered standard-dose apixaban [3, 5], which is consistent with the univariate analysis results in the present study. In this study, the difference in mortality between patients taking standard- and low-dose apixaban disappeared after adjusting for clinically relevant factors or when stratified by age (80 years). This finding suggests that underlying factors such as age are responsible for the increased mortality observed in patients taking low-dose apixaban.

The strengths of this study include the large registry of acute ischemic stroke due to LVO and AF and the use of a single DOAC for stroke prevention. However, this study had several limitations. First, although the patients of this study received apixaban as instructed, the rate of inappropriately high or low doses of apixaban could not be analyzed because the ALVO study, which primarily planned to confirm the safety of early administration of apixaban, did not collect body weight data. Several real-world studies demonstrated that 25–36% of patients were administered receiving inappropriately doses of apixaban [2, 4, 19]. The results of this study reflect the real-world practice but not reflect the on-label use of apixaban. Second, the possibility of residual confounding by unmeasured factors such as frailty status or body weight still exists. Several studies reported that atrial fibrillation in frail or low body weight patients was associated with a higher risk of all-cause death, ischemic stroke, and bleeding [20–23]. Patients eligible for apixaban dose reduction also had a higher prevalence of frailty or low body weight, which may have influenced the outcomes. Third, the choice of apixaban and the timing of apixaban initiation after ischemic stroke were at the discretion of the treating physician. It is possible that some patients had a recurrent stroke before starting apixaban and were excluded from the study. In this study, the median days from onset to apixaban administration were 3.5 and 4 days in the standard- and low-dose apixaban groups, respectively, which is comparable to the most recent recommendation [24]. Finally, the findings of this study cannot be generalized to other population groups or DOACs. Each DOAC has different criteria for dose adjustment; thus, evaluating the efficacy and safety of multiple low-dose DOACs is difficult.

## Conclusion

No statistically significant differences were observed in the incidence of ischemic events, major bleeding events, or death from any cause between patients with AF after LVO stroke who received standard-dose apixaban and those who received low-dose apixaban.

### Supplementary Information

Below is the link to the electronic supplementary material.

Supplementary file1 (PDF 157 kb)

## Data Availability

The data that support the findings of this study are available from the corresponding author upon reasonable request.
