# Supplementary material for: One-year morbidity and mortality in patients treated with standard-dose and low-dose apixaban after acute large vessel occlusion stroke
Source: J Thromb Thrombolysis. 2024 Mar 31;57(4):622–9. doi: 10.1007/s11239-024-02954-7 (PMC11026242; doi:10.1007/s11239-024-02954-7)
Supplement: Supplementary file 1 — Supplementary file1 (PDF 157 kb) [file 11239_2024_2954_MOESM1_ESM.pdf]

Supplementary Materials

Full Title:

One-year morbidity and mortality in patients treated with standard-dose and low-dose apixaban after acute large vessel occlusion stroke

Supplemental Table 1

Crude hazard ratios for ischemic events in patients administered low-dose apixaban compared to patients with standard-dose apixaban

Subgroup		Standard dose n (%/year)	Low dose n (%/year)	Crude HR (95% CI)	p for interaction
Sex	male	8 (4.0)	6 (7.7)	1.86 (0.64–5.36)	0.38
	female	1 (1.1)	9 (6.1)	5.32 (0.67–42.1)	
Age	≥80	3 (9.0)	12 (6.7)	0.73 (0.20–2.57)	0.16
	<80	6 (2.3)	3 (6.5)	2.76 (0.69–11.0)	
Initial treatment with IV rt-PA	Yes	3 (2.1)	5 (6.3)	2.76 (0.66–11.6)	0.54
	No	6 (4.0)	10 (6.8)	1.65 (0.60–4.55)	
Initial treatment with EVT	Yes	3 (1.7)	10 (8.3)	4.64 (1.28–16.9)	0.060
	No	6 (5.2)	5 (4.8)	0.89 (0.27–2.92)	

Abbreviation: CI, confidence interval; EVT, endovascular therapy; NIHSS, National Institute of Health Stroke Scale; HR, hazard ratio; IV rt-PA, intravenous recombinant tissue plasminogen activator

Supplemental Table 2

Crude hazard ratios for major bleeding events in patients administered low-dose apixaban compared to patients with standard-dose apixaban

Subgroup		Standard dose n (%/year)	Low dose n (%/year)	Crude HR (95% CI)	p for interaction
Sex	male	15 (7.6)	8 (10.4)	1.29 (0.55–3.05)	>0.99
	female	4 (4.4)	9 (6.1)	1.29 (0.40–4.19)	
Age	≥80	5 (15.6)	12 (6.7)	0.41 (0.15–1.18)	0.036
	<80	14 (5.4)	5 (10.9)	1.96 (0.71–5.44)	
Initial treatment with IV rt-PA	Yes	7 (4.9)	5 (6.2)	1.22 (0.39–3.85)	0.75
	No	12 (8.2)	12 (8.3)	0.94 (0.42–2.10)	
Initial treatment with EVT	Yes	12 (6.9)	11 (9.2)	1.26 (0.55–2.85)	0.60
	No	7 (6.1)	6 (5.8)	0.87 (0.29–2.60)	

Abbreviations: CI, confidence interval; EVT, endovascular therapy; NIHSS, National Institute of Health Stroke Scale; HR, hazard ratio; IV rt-PA, intravenous recombinant tissue plasminogen activator

Supplemental Table 3

Crude hazard ratios for death from any cause in patients administered low-dose apixaban compared to patients with standard-dose apixaban

Subgroup		Standard dose n (%/year)	Low dose n (%/year)	Crude HR (95% CI)	p for interaction
Sex	male	6 (2.9)	11 (13.6)	4.65 (1.72–12.6)	0.90
	female	3 (3.2)	21 (13.9)	4.28 (1.28–14.3)	
Age	≥80	3 (8.9)	30 (16.3)	1.81 (0.55–5.91)	0.99
	<80	6 (2.3)	2 (4.2)	1.87 (0.38–9.29)	
Initial treatment with IV rt-PA	Yes	2 (1.4)	8 (9.7)	7.14 (1.52–33.7)	0.43
	No	7 (4.6)	24 (16.0)	3.47 (1.49–8.05)	
Initial treatment with EVT	Yes	7 (3.9)	13 (10.3)	2.67 (1.06–6.64)	0.11
	No	2 (1.7)	19 (18.0)	10.3 (2.41–44.4)	

Abbreviations: CI, confidence interval; EVT, endovascular therapy; NIHSS, National Institute of Health Stroke Scale; HR, hazard ratio; IV rt-PA, intravenous recombinant tissue plasminogen activator

Supplemental Table 4

Cox regression derived hazard ratios and 95% confidence intervals for ischemic events

	Crude HR (95% CI)	p-value	Adjusted HR (95% CI)	p-value
Low-dose apixaban	2.06 (0.90–4.72)	0.086	1.95 (0.85–4.46)	0.12
Age (per 1 year)	1.03 (0.98–1.08)	0.22		
Female sex	0.81 (0.36–1.83)	0.61		
Hypertension	1.03 (0.41–2.59)	0.96		
Diabetes mellitus	3.21 (1.10–9.38)	0.033	2.99 (1.02–8.78)	0.046
Chronic heart failure	1.40 (0.33–5.94)	0.65		
History of ischemic stroke	1.72 (0.68–4.34)	0.25		
Prior antiplatelet drug	0.61 (0.18–2.04)	0.42		
Prior anticoagulation	2.80 (1.25–6.25)	0.012	2.57 (1.15–5.75)	0.022
Creatinine (per 1 mg/dl)	1.85 (0.59–5.86)	0.29		
Initial treatment with IV rt-PA	0.68 (0.29–1.60)	0.38		
Initial treatment with EVT	0.88 (0.40–1.97)	0.76		

Abbreviations: CI, confidence interval; EVT, endovascular therapy; HR, hazard ratio; IV rt-PA, intravenous recombinant tissue plasminogen activator

Supplemental Table 5

Cox regression derived hazard ratios and 95% confidence intervals for bleeding events

	Crude HR (95% CI)	p-value	Adjusted HR (95% CI)	p-value
Low-dose apixaban	1.08 (0.56–2.07)	0.82	0.97 (0.50–1.88)	0.92
Age (per 1 year)	1.00 (0.97–1.04)	0.80		
Female sex	0.64 (0.32–1.26)	0.19		
Hypertension	2.03 (1.04–3.96)	0.039	1.98 (0.98–3.99)	0.058
Diabetes mellitus	3.21 (1.34–7.71)	0.009	2.21 (0.87–5.62)	0.095
Chronic heart failure	1.84 (0.65–5.22)	0.25		
History of ischemic stroke	3.02 (1.53–5.96)	0.001	2.83 (1.38–5.80)	0.004
Prior antiplatelet drug	2.20 (1.10–4.39)	0.026	1.52 (0.73–3.20)	0.26
Prior anticoagulation	1.45 (0.71–2.94)	0.31		
Creatinine (per 1 mg/dl)	1.88 (0.72–4.92)	0.20		
Initial treatment with IV rt-PA	0.67 (0.34–1.34)	0.26		
Initial treatment with EVT	1.35 (0.68–2.66)	0.39		

Abbreviations: CI, confidence interval; EVT, endovascular therapy; HR, hazard ratio; IV rt-PA, intravenous recombinant tissue plasminogen activator

Supplemental Table 6

Cox regression derived hazard ratios and 95% confidence intervals for death from any cause

	Crude HR (95% CI)	p-value	Adjusted HR (95% CI)	p-value
Low-dose apixaban	4.55 (2.16–9.53)	<0.001	2.00 (0.81–4.93)	0.13
Age (per 1 year)	1.10 (1.06–1.15)	<0.001	1.07 (1.01–1.12)	0.018
Female sex	1.64 (0.88–3.06)	0.12		
Hypertension	0.87 (0.41–1.81)	0.70		
Diabetes mellitus	0.77 (0.19–3.21)	0.72		
Chronic heart failure	2.69 (1.13–6.39)	0.025	1.83 (0.74–4.52)	0.19
History of ischemic stroke	2.39 (1.24–4.61)	0.010	1.91 (0.97–3.76)	0.063
Prior antiplatelet drug	1.81 (0.92–3.54)	0.085		
Prior anticoagulation	1.50 (0.78–2.89)	0.23		
Creatinine (per 1 mg/dl)	1.75 (0.73–4.20)	0.21		
Initial treatment with IV rt-PA	0.43 (0.21–0.88)	0.021	0.57 (0.28–1.16)	0.12
Initial treatment with EVT	0.70 (0.38–1.29)	0.25		

Abbreviations: CI, confidence interval; EVT, endovascular therapy; HR, hazard ratio; IV rt-PA, intravenous recombinant tissue plasminogen activator

Supplemental Table 7

Study investigators

	Institutes	Investigators
**1**	Kobe City Medical Center General Hospital	Shinichi Yoshimura
**2**	Nishinomiya Kyouritsu Neurosurgical Hospital	Kotaro Tatebayashi
**3**	Seisho Hospital	Masataka Takeuchi
**4**	National Cerebral and Cardiovascular Center	Kazunori Toyoda
**5**	Iwate Prefectural Central Hospital	Naoto Kimura
**6**	Yoshida Hospital.Cerebrovascular Research Institute	Ikuya Yamaura
**7**	Sapporo Shiroishi Memorial Hospital	Tadashi Nonaka
**8**	Showa University Koto Toyosu Hospital	Yuki Kamiya
**9**	Hirosaki University	Hiroki Okuma
**10**	Kobe City Medical Center General Hospital	Nobuyuki Sakai
**11**	Yokohama Shintoshi Neurosurgical Hospital	Masafumi Morimoto
**12**	Goshi Hospital	Yoshiharu Oki
**13**	Sato Daiichi Hospital	Shigehiro Nakahara
**14**	Kokura Kinen Hospital	Taketo Hatano
**15**	Gifu University	Yukiko Enomoto
**16**	Fukuoka University Chikushi Hospital	Kohei Nii
**17**	Hakodate Shintoshi Hospital	Koichi Haraguchi
**18**	Kurashiki Central Hospital	Akira Handa
**19**	National Hospital Organization Osaka Minami Medical Center	Junya Kobayashi
**20**	Japan Community Health Care Organization Kobe Central Hospital	Keigo Matsumoto
**21**	Mie University Hospital	Naoki Toma
**22**	MAZDA Hospital	Ryo Ogami
**23**	Shimizu Hospital	Fuminori Shimizu
**24**	Kyoritsu Hospital	Tomoko Iida
**25**	Kindai University	Amami Kato
**26**	Miyakonojo Medical Association Hospital	Shunro Uchinokura
**27**	Tokyo Metropolitan Tama Medical Center	Takahiro Ota
**28**	Ube-kohsan Central Hospital	Norio Ikeda
**29**	Japanese Red Cross Kyoto Daiichi Hospital	Keisuke Imai
**30**	Osaka University Hospital	Kenichi Todo

**31**	Kagawa University Hospital	Atsushi Shindo
**32**	Hirosaki Stroke and Rehabilitation Center	Joji Hagii
**33**	Kyoto University	Akira Ishi
**34**	Yamaguchi Prefectural Grand Medical Center	Hiroaki Yasuda
**35**	Sanda City Hospital	Akinori Nose
**36**	Kinki Central Hospital	Junji Ueda
**37**	Toranomon Hospital	Wataro Tsuruta
**38**	Japanese Red Cross Nagoya Daini Hospital	Keizo Yasui
